# Genetic barcoding of museum eggshell improves data integrity of avian biological collections

**DOI:** 10.1038/s41598-020-79852-4

**Published:** 2021-01-15

**Authors:** Alicia Grealy, Naomi E. Langmore, Leo Joseph, Clare E. Holleley

**Affiliations:** 1 Langmore Group, Research School of Biology, Australian National University, Building 46, Canberra, 0200 Australia; 2 Australian National Wildlife Collection, National Research Collections Australia, CSIRO, Canberra, 2601 Australia

**Keywords:** Genetic techniques, Sequencing, Evolutionary biology, Genotype, Sequencing, Evolutionary genetics, Palaeontology, Taxonomy, Conservation biology, Biological techniques, Ecology, Evolution, Genetics

## Abstract

Natural history collections are often plagued by missing or inaccurate metadata for collection items, particularly for specimens that are difficult to verify or rare. Avian eggshell in particular can be challenging to identify due to extensive morphological ambiguity among taxa. Species identifications can be improved using DNA extracted from museum eggshell; however, the suitability of current methods for use on small museum eggshell specimens has not been rigorously tested, hindering uptake. In this study, we compare three sampling methodologies to genetically identify 45 data-poor eggshell specimens, including a putatively extinct bird’s egg. Using an optimised drilling technique to retrieve eggshell powder, we demonstrate that sufficient DNA for molecular identification can be obtained from even the tiniest eggshells without significant alteration to the specimen’s appearance or integrity. This method proved superior to swabbing the external surface or sampling the interior; however, we also show that these methods can be viable alternatives. We then applied our drilling method to confirm that a purported clutch of Paradise Parrot eggs collected 40 years after the species’ accepted extinction date were falsely identified, laying to rest a 53-year-old ornithological controversy. Thus, even the smallest museum eggshells can offer new insights into old questions.

The world’s natural history collections contain ca. 5 million specimens of avian eggshells^1^ (with more than one million housed at the Natural History Museum at Tring, UK, alone^2^), that can be used to address a myriad of otherwise intractable questions^3,4^. However, the identity of such specimens often relies on the accuracy of the collector’s metadata; many eggshell specimens without metadata cannot be identified to species and therefore currently have little utility for research. Additionally, there have been cases of fraud and sensationalism where private egg collectors have been motivated to make claims about the occurrence of rare, threatened, endangered and even sometimes ‘extinct’ species^5–7^. Thus, to fully realise the scientific potential of the vast repository of avian biodiversity data held in egg collections, we need to improve the confidence of species identification in eggs.

Recently, Grealy et al.^8^ demonstrated that DNA extracted from just 10 mg of museum eggshell can be used to identify species through the amplification and sequencing of molecular ‘barcodes’. However, the success of that methodology has only been demonstrated on three medium-to-large sized eggshells weighing > 10 g and with a thickness > 0.4 mm. As many eggshell specimens are significantly smaller than this, it remains uncertain whether very small eggshells can be sampled for DNA without incurring significant damage to the specimen. Here, we have further optimised the methods of Grealy et al.^8^ for use with small museum eggshells, setting a precedent for molecular studies of even the smallest eggs in museum collections world-wide. We amplified and sequenced two *12S rDNA* mitochondrial mini-barcodes^9^ from DNA extracted from variously-sized, unidentified museum eggshell specimens using three sampling methodologies, and compared the genetic profiles obtained from each approach: (a) a swab from the external surface of the eggshell; (b) a sample of the internal surface, taken via injection of water into the internal cavity, and (c) eggshell powder collected by widening the existing blow hole via drilling. We then applied the best of these methods to demonstrate how it can be used to clarify a long-standing, contentious debate in Australian ornithology without compromising the integrity of a potentially rare and irreplaceable museum eggshell specimen.

To hone our technique and qualitatively determine the factors (such as egg size and thickness) that influenced the viability of our sampling protocol, we documented the damage (SI 1.1) that each sampling strategy (SI 1.2; Supplementary Fig. S1) incurred to 51 otherwise unidentifiable, unregistered eggshell specimens, many already sporting physical damage (Supplementary Table S1). These specimens were chosen because most museums are reluctant to loan registered collection items for unverified experimental work. We found that while the shape and size of the drill bit used to drill eggs was critical (Supplementary Fig. S2), pre-existing damage (cracks, chips, hair-line fractures) was the clearest predictor of further eggshell breakage rather than size or thickness, when using the optimal drill bit. External swabs were able to be collected from all eggs regardless of size or pre-existing damage. However, pre-existing damage to the egg was detrimental to sampling via both drilling and buffer injection when the eggshell weighed less than 0.1 g: vibrations from the drill caused hair-line fractures to grow, and the large surface area-to-volume ratio of small eggs caused water to strongly adhere to the internal surface, making it near-impossible to drain the water without rough handling. Larger eggs (ca. 0.3 g or more) could be drilled without incurring additional damage, regardless of their pre-existing condition. Eggs with blow holes drilled in the poles as opposed to the midline were also more fragile; in some instances, drilling a new lateral hole proved less detrimental to the specimen’s overall structure than expanding an existing hole at either pole.

For DNA extraction (SI 1.3) and barcoding (SI 1.4), we selected a separate set of 35 intact, unregistered and (mostly) unidentified eggshell specimens weighing between 0.052–3.299 g, and sampled these using the methods above (SI 1.2). Even for the smallest eggs, our drilling strategy had little impact on the overall integrity of the specimen, as the diameter of the existing blow hole was extended by (on average) 1.39 mm (1.27 × the initial diameter), and to no more than 5 mm maximum regardless of the egg’s size (Fig. 1; Supplementary Table S2). Once the drilling strategy proved viable, we applied it to clarify the identity of an additional 10 registered but unidentified eggs, including one controversially classified as Paradise Parrot (*Psephotellus pulcherrimus*), an Australian bird 92 years extinct that laid a nondescript white egg.

**Figure 1:**
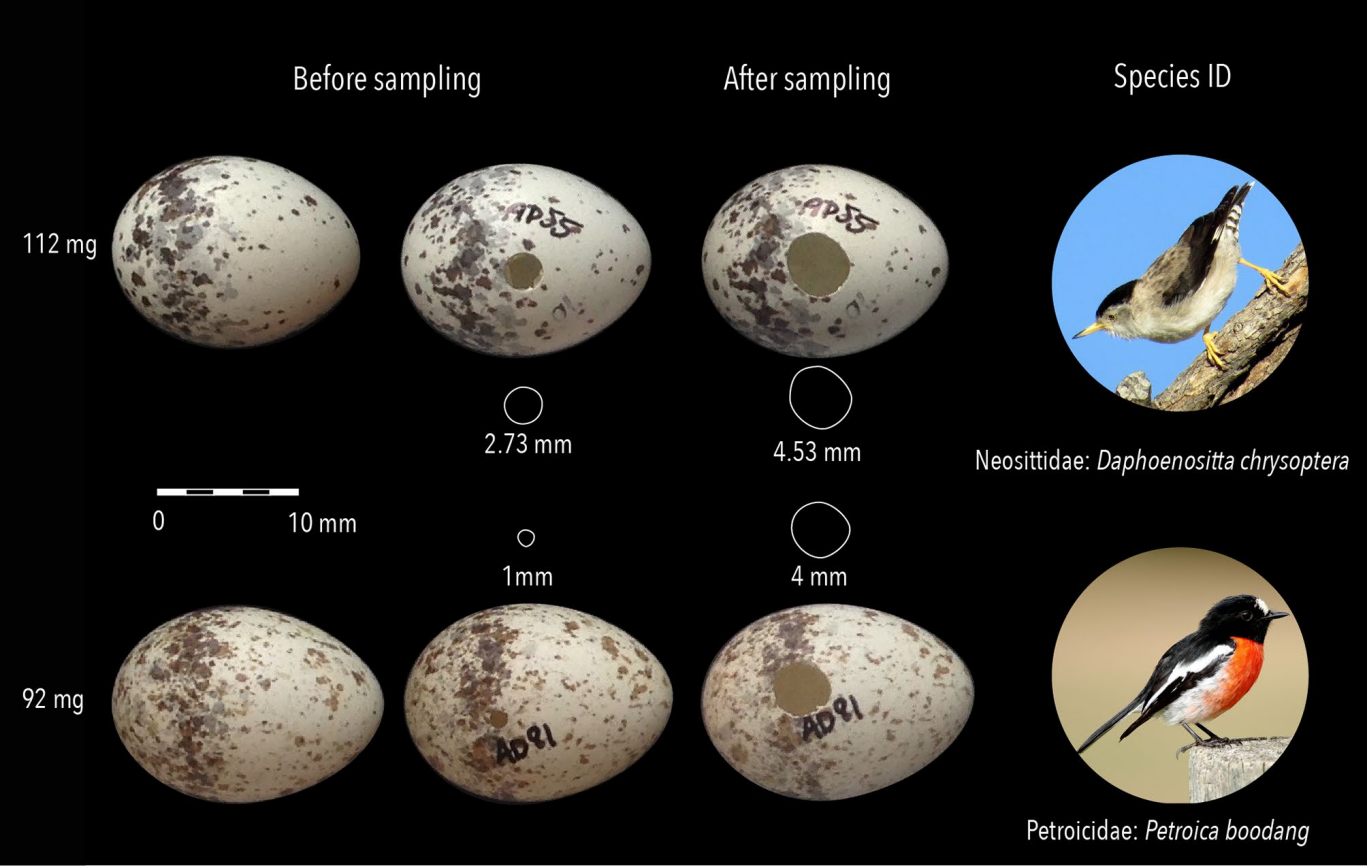
Examples of unidentified eggshell that were drilled for DNA extraction. Note these eggs are similar morphologically yet DNA mini-barcodes suggest they belong to different species, and indeed, different families. Also of note is that these eggs are both less than 2 cm long and weigh ca. 100 mg, yet positive IDs were obtained from both with minor damage incurred to the specimen by sampling (the diameter of the blow hole prior to and after sampling are indicated). Bird photographs taken by Graham Winterflood, supplied to the *Atlas of Living Australia*^22^ by *iNaturalist Australia* and *BowerBird.org.au*, and reproduced under the Creative Commons license.

We successfully extracted (SI 1.3) and amplified a *12SrDNA* mitochondrial mini-barcode 100 base pairs (bp) in length (“12SAC”) from the powder of all these specimens, and were able to amplify a 250 bp *12S* mitochondrial barcoding region from 69% (“12SAH”^9^; SI 1.4). This *12SrDNA* locus has been used for species identification of illegally smuggled bird’s eggs^10^, and allows us to gauge the fragment-length of amplifiable DNA because both a longer and shorter amplicon spans this region. Traditional barcodes such as *CO1* rely on the presence of long fragments (ca. 600 bp) that are not typically recovered from historic or environmental samples, and *12S* is therefore often more suitable for the amplification of degraded DNA^9,11,12^. The failure of 12SAH to amplify in some specimens suggests these had DNA degraded to less than 250 bp in length. Amplification was not correlated with the size or thickness of the egg for either barcode (*p* > 0.15; SI 1.6). For each sample, we sequenced the longest barcode that amplified on Illumina’s MiSeq (single-end, Nano 300 cycle v2 kit) (SI 1.4), each to a depth of approximately 6,500 reads (total 842,086 reads). For those samples that were able to be sequenced, 90.9% of powder extracts had 12SAC reads pass quality control (SI 1.5), whereas 67.7% had 12SAH reads pass (as a longer amplicon, it is more prone to accumulating PCR and sequencing error). On average, 12SAC yielded 1.5 filtered, unique reads per powder extract, while 12SAH yielded two. Typically, for powder samples that yielded more than one unique read, the less-abundant reads accounted for below 5%, whereas the most abundant read accounted for over 90% of the reads. The less-abundant reads likely correspond to within-species genetic variation (returning the same ID as the most abundant read), or represent low-level contamination, returning an impossible ID (e.g., chicken *Gallus gallus*) (Supplementary Table S4).

Molecular IDs were ultimately deemed plausible if the egg morphology of the specimen appeared consistent with published reference photographs of the taxon from the *Atlas of Living Australia*. Comparison of the most abundant filtered, unique sequences with GenBank’s nucleotide database (November, 2019) via BLASTn (SI 1.5) provided plausible species-level identifications (IDs) for 6/45 specimens (13.3%), 18/45 genus-level IDs (40%), 9/45 family-level IDs (20%), and 10/45 order-level IDs (22.2%), with just two specimens (4.4%) yielding no ID (Supplementary Table S2). From the powder, both amplicons yielded consistent IDs, except for one sample where it was the second most abundant 12SAH read that provided the plausible ID consistent with 12SAC. There was no correlation between the size of the egg or eggshell thickness on any metric measured (i.e., reads pass filter, plausibility of ID, resolution of ID). The resolution of identification was hindered by the incompleteness of the reference database for *12S*, and the lack of barcode gap to distinguish certain high biodiversity groups, such as Australasian honeyeaters (Meliphagidae), where many different genera are identical across this short segment of *12S*. It should be a priority to expand the reference database, both for these barcodes, as well as others. Barcodes could be selected based on *in-silico* analysis of reference database completeness for the taxonomic group(s) of interest, and multiple barcodes could be used to corroborate identifications. Longer barcodes (such as *CO1*) may be able to be reconstructed by amplifying multiple, smaller overlapping fragments. Nevertheless, the fact that mitochondrial fragments of at least 100 bp can be amplified suggests that complete mitogenomes could be reconstructed from the extracted DNA^8^. Thus, the crux of the matter is that species identification of small eggshells genetically is limited by the inclusiveness of the reference database rather than an inability to recover DNA of sufficient quality.

As with the powder, 12SAC barcodes also amplified from all the corresponding swabs and internal samples, while 12SAH amplified with less success: 61.3% amplified for swabs, while the majority failed to amplify from the internal samples (55.6%). Normalised to a positive control of modern, high-quality DNA, powder and swabs contained on average over 30X more copies of the endogenous target templates than the internal samples for 12SAC, and over 9X more for 12SAH. Furthermore, for both amplicons, swabs and internal samples had fewer extracts pass filter compared with the powder (12SAC: 77.3% for swabs, 71.4% for internal; 12SAH: 63.2% for swabs, 75% for internal). Those sequences that did pass filter generated profiles that were in general not consistent between barcodes: in these cases, 12SAH was the amplicon producing the implausible ID. This is probably because contamination is typically longer in fragment length, so where endogenous DNA is degraded, contamination will amplify instead. Comparing profiles between extract types, in cases where all three amplified, only 38.5% generated IDs in agreement with one another (Supplementary Fig. S3).

As expected, drilling eggshell was most successful at recovering a plausible species ID for both barcode amplicons and should therefore be considered best practice (Fig. 2). Powder consistently outperformed swabs and internal samples in every way: it produced the greatest quantity and quality of DNA and consequently, the most unambiguous and consistent molecular IDs. This was expected because DNA trapped in the intra-crystalline matrix of the eggshell is largely protected from damage, decay and contamination^13^. However, many small eggs can only be drilled once, so curators will need to be conservative in their assessments of when a specimen should be sampled, if at all. Of course, sampling eggs that are already fragmented is the least risky of all, and such eggs should remain in collections for this purpose if nothing else.

**Figure 2:**
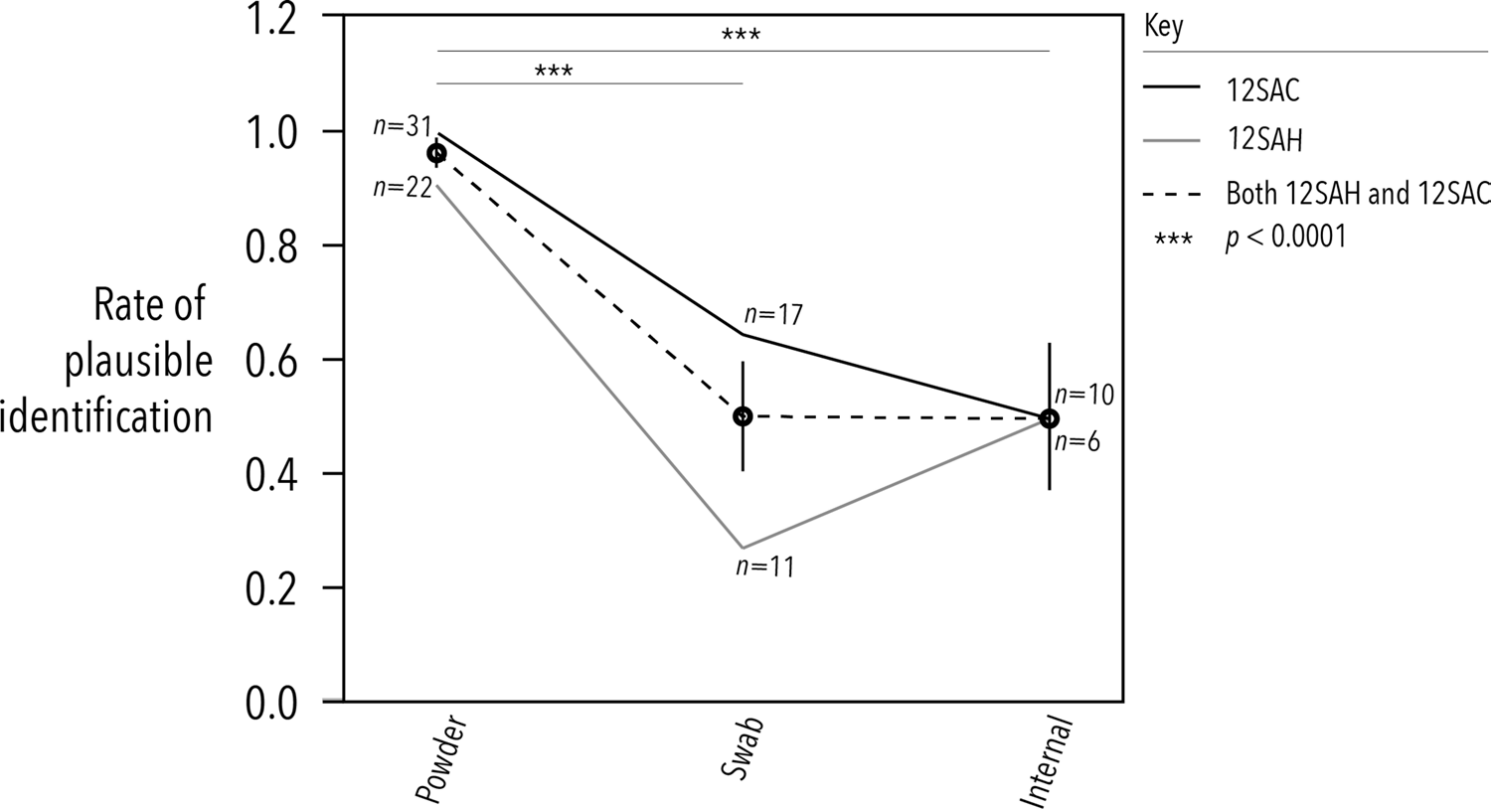
The rate of plausible molecular identifications for each barcode by extract type for the most abundant read sequenced. The criteria for assigning a molecular identity are detailed in SI 1.5. Note that sample sizes differ because some extracts failed to amplify or produced poor quality sequences that were discarded during quality control. For a complete list of molecular identifications for all 45 eggshell specimens sampled for DNA by substrate and barcode, see Supplementary Table S2. Intervals indicate 95% confidence on the standard error of the mean. Statistical significance is based on pair-wise Mann–Whitney tests with a Bonferroni correction.

Unexpectedly, the less invasive fluid sampling and non-destructive swab approach did return plausible species IDs about 50% of the time (Fig. 2), but were also more susceptible to contamination because of their reliance on surface DNA. Several unique sequences belonging to different taxa were obtained from the swab (on average 12.3 unique reads for 12SAC and 1.9 unique reads for 12SAH) and internal samples (on average 2.2 unique reads for 12SAC and 1.5 unique reads for 12SAH), and often these taxa could only be considered contamination because they were conspicuously inconsistent with the morphology of the egg, or had very few reads (Supplementary Table S4). Extraneous sequences amplified from swabs and internal samples (and to a lesser extent powder) probably originate from cross-contamination when handling (either during collection, curation, or study), rather than sequencing error which should be filtered out during quality control. This highlights the importance of using personal-protective equipment when handling collection specimens to reduce contamination. Thus, while the low or non-invasive approaches are less reliable and accurate, they could be used cautiously for species ID in damaged or high-value specimens that cannot be drilled. However, one must be cognisant that swabbing may remove surface colouration in some specimens, or the crucially important collector’s ‘set marks’, which are part of the specimen’s metadata. Certainly though, where eggs are being cleaned anyway, preparators should keep these swabs for posterity. Though DNA can be retrieved from the membranes of museum eggshell by removing them through the blow hole^14^, it may be unmanageable to extract membranes from very small eggs. Thus, while these methods may all be viable alternatives, DNA extracted from powder remains more amenable to whole mitogenome sequencing and possible nuclear sequencing^8^. Thus, it would be more ideal for analysis of population-level genetic diversity that could lead to re-evaluations of evolutionary significant units (ESUs), species boundaries, or evolutionary history within avian taxa.

Lastly, we used our optimised drilling protocol to resolve the species identification of one egg specimen from a contentiously identified clutch of eggs within the Australian National Wildlife Collection, Canberra (ANWC E10619; Fig. 3a), providing an example of how our method can improve data integrity in biological collections. We extracted DNA from eggshell powder sampled from an egg putatively of the paradise parrot (*Psephotellus pulcherrimus*; Fig. 3b), expanding the diameter of the blow hole by only half a millimetre (Fig. 3a). This egg came from a clutch collected by R. Guyatt in Toobeah, south-east Queensland (Australia) in 1967, and had been hailed as the rediscovery of a species that had reportedly become extinct in 1927–1928^15,16^; thus, it was of significant interest for conservation research. The accuracy of the species ID for these putative paradise parrot eggs has been questioned^15^, but the matter could not be unambiguously resolved with morphology because the paradise parrot, like all parrots, laid a nondescript white egg devoid of markings, making the egg difficult to distinguish from eggs of other similar-sized parrots. The identity of this clutch remains controversial because the eggs are unusually large compared with confirmed paradise parrot eggs; the largest known paradise parrot egg measures 23 × 20 mm while the Guyatt eggs average 26 × 21 mm^15^. While the sampled egg measures 25.76 × 21.21 mm (Fig. 3a), the smallest of the Guyatt eggs belong to the same clutch^15^. Olsen^15^ argued that these eggs are too large for any species in the genus *Psephotellus*, though as a “notoriously variable characteristic”^17^, size alone cannot be used to definitively reject the claim. If this clutch is truly of the paradise parrot, it would push the date of their extinction forward by some 40 years. DNA testing of the Guyatt eggs offers perhaps the only opportunity to finally settle the issue^17^.

**Figure 3:**
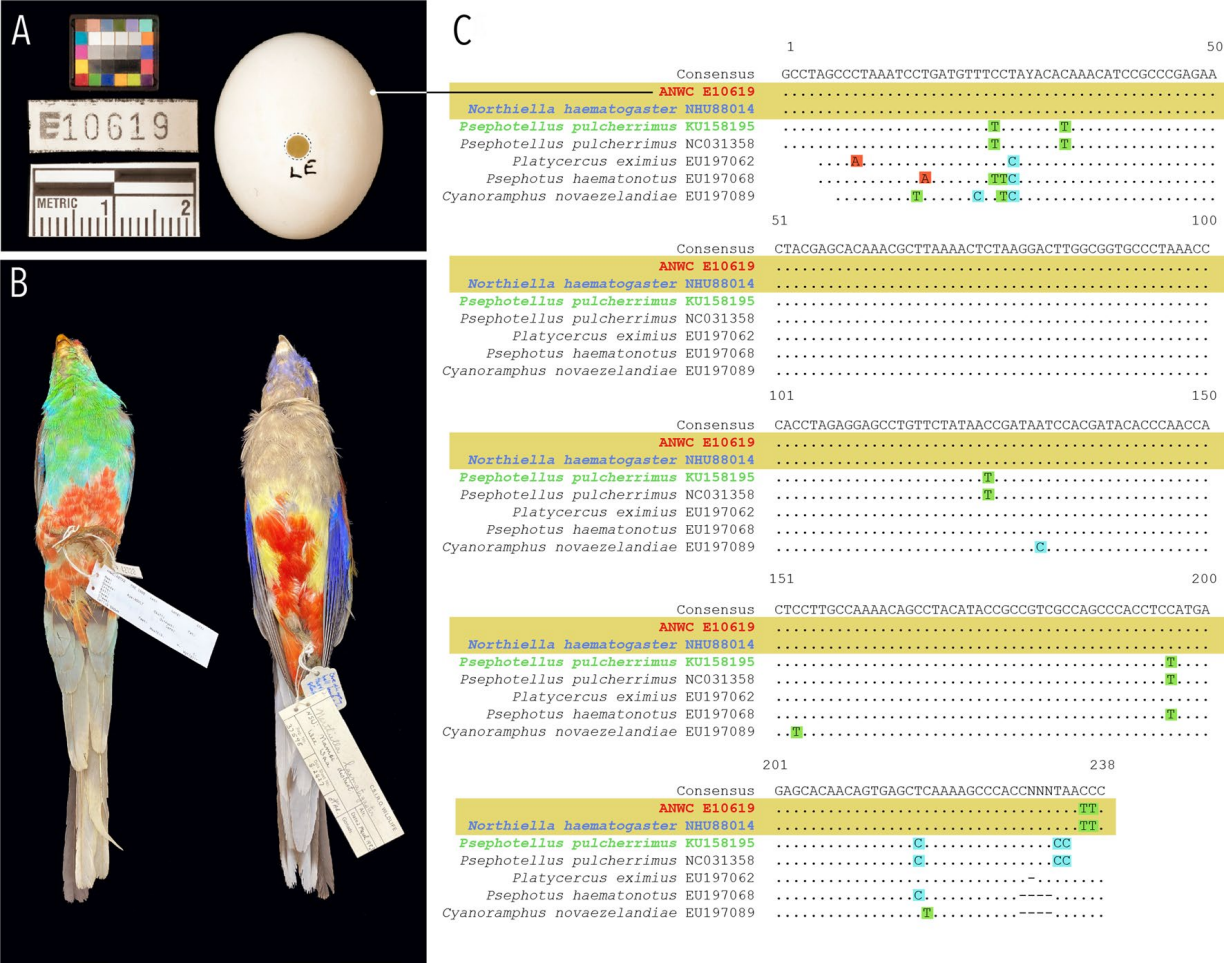
(**A**) photograph of putative paradise parrot (*Psephotellus pulcherrimus*) egg. The dotted line around the blow hole represents the size of the blow hole after drilling. (**B**) Image of specimens in the Australian National Wildlife Collection of the extinct paradise parrot (left) alongside an adult eastern bluebonnet (*Northiella haematogaster*, right), the species to which the disputed egg (in **A**) actually belongs. (**C**) An alignment of the 12SAH barcode sequenced from the eggshell specimen in question alongside genetic references from members of the tribe Platycercini. Dots represent bases identical to the consensus, and yellow shading highlights the reference sequence that is 100% identical to the eggshell specimen sequence.

We compared the sequence obtained from the amplification of both 12SAC and 12SAH barcodes to NCBI’s GenBank reference database using BLASTn (SI 1.5). Both barcodes were 100% identical across their entire lengths to the extant and still common eastern bluebonnet, *Northiella haematogaster* (Fig. 3b), whereas sequence identity was only 94% similar to the paradise parrot (Fig. 3c). Although not all genera within the family Psittaculidae (sensu Joseph et al.^18^) are represented by a genetic reference for this *12S* locus, based on those that are, it is unlikely that the sequence in question could belong to a different species not represented in GenBank: the average identity within species across the 12SAH barcode is 99.6%, while the average identity between species within genera is 96.7%, and the average identity between genera within the family is 93.1%. (Supplementary Table S3). Further, *Psephotellus* is most closely related to monotypic *Purpureicephalus*, and that pair of genera is in turn closest to *Northiella* and monotypic *Psephotus*^19,20^—all of which are represented in GenBank for *12S*. Therefore, we could infer that it would be even more distant from the other genera, even though we do not have a *12S* reference for them. Thus, we conclude that the egg is not of the paradise parrot but rather of a parrot within the extant genus *Northiella*, most likely *N. haematogaster* (rather than *N. narethae*) based on species distributions and the high sequence identity to the reference. Supporting our results, Olsen^15^ suggested *N. haematogaster* among the most likely candidates for the true identity of the egg and its associated clutch. This case of mistaken identity demonstrates the value of our molecular method to evaluate and test the veracity of metadata held within historical egg collections and facilitates their use as a trusted source of avian biodiversity data.

Museum specimens provide unparalleled opportunities to examine how diversity has changed across time. Museum eggshells in particular are an untapped resource of genomic information for thousands of avian species, including rare and extinct taxa^1^. The methods presented here provide a resource for other collections around the world to perform genetic analysis on eggshells in a cost-effective manner with minimal impact on the appearance of the specimen. This introduces new possibilities to interrogate biological phenomena, such as coevolution between brood parasites and their hosts. Furthermore, our method of sampling eggshell powder could potentially be used for other techniques such as mass spectrometry, stable isotope analysis, protein fingerprinting, whole-genome sequencing, or even transcriptomics; these techniques have applications in the study of reproductive biology, metabolism, avian diet, ecology, egg forensics^10^ and evolutionary history. It may also be a promising way to non-destructively extract DNA from the calcareous shells of some reptile eggs, as well as mollusc exoskeletons^21^, which are often abundant among archaeological assemblages or serve as biological indicators of ecosystem health.

These methods can improve the curation and scientific value of collections by providing high-confidence taxonomic identifications and additional occurrence records for species that can help clarify the historical range and distribution of threatened and endangered species. Such information may, in turn, improve biodiversity assessments that could inform conservation management and the designation of protected areas.

## Supplementary information

Supplementary Information 1

Supplementary Information 2

Supplementary Information 3

Supplementary Information 4

## Data Availability

The DNA sequences generated have been deposited at DataDryad at 10.5061/dryad.k3j9kd55x. Correspondence and requests for material should be addressed to AG (alicia.grealy@uqconnect.edu.au).
