# Supplementary material for: Genetic barcoding of museum eggshell improves data integrity of avian biological collections
Source: Sci Rep. 2021 Jan 15;11:1605. doi: 10.1038/s41598-020-79852-4 (PMC7810714; doi:10.1038/s41598-020-79852-4)
Supplement: Supplementary file 1 — Supplementary Information 1 [file 41598_2020_79852_MOESM1_ESM.pdf]

Supplementary Information

Genetic barcoding of museum eggshell improves data integrity of avian biological collections

Alicia Grealy[1,2*], Naomi Langmore[1], Leo Joseph[2], Clare Holleley[2]

[1] Langmore Group, Research School of Biology, Building 46, Australian National University, Canberra, ACT 0200, Australia

[2] Australian National Wildlife Collection, National Research Collections Australia, CSIRO, Canberra, ACT 2601, Australia

* Corresponding author. E-mail: alicia.grealy@uqconnect.edu.au

ORCIDs for authors: Clare Holleley 0000-0002-5257-0019, Alicia Grealy 0000-0003-1533-0144

Supplementary Information

SI 1.0 Supplementary Methods

SI 1.1 Characterisation collection eggshell (mass, thickness, damage)

Eggshell specimens were weighed on an electronic balance, and the diameter of the blow hole was measured (Table S1, Table S2). The thickness of eggshell at the egg apex, and around the meridian were measured using a magnetic thickness gauge (MiniTest7200FH) with a 1.5 mm metallic ball, following the methods described by Grieve et al. (*in preparation*) (Table S1). Eggshell was illuminated under a goose-neck cold light source to check for hairline fractures. After drilling (SI 1.2), the diameter of the blow hole was measured again, and any further damage to the eggshell was recorded (Table S1).

SI 1.2 Sampling collection eggshell for DNA extraction

Note that eggshell specimens were not cleaned in any way prior to sampling. As we aimed to recover DNA profiles from the surface, we avoided UV irradiation or bleaching (which is often used to remove surface contamination from ancient specimens). Furthermore, many specimens have patterns that can be removed by bleaching or UV irradiation, and when sampling museum specimens non-destructively, this must be avoided at all costs.

Water was used for the steps below as it was unknown how other buffers would react with the eggshell in the short or long-term, potentially impacting the longevity of the specimen. A buffer such as PBS could be tested in the future.

All sampling steps were carried out in a designated ultra-clean facility at the ANU (Ecogenomics and Bioinformatics Laboratory) inside a designated UV hood. Tools, racks and working surfaces were cleaned with 10% house-hold bleach followed by 70% ethanol, and tools and racks were UV irradiated in a UV sterilising cabinet for 20 minutes. Full coveralls with an additional disposable apron, face mask, eye goggles, rubber boots with disposable booties, and two pairs of nitrile gloves were worn at all times. Gloves were wiped over with bleach changed along with the aprons between samples.

Surface swabs. A sterile cotton swab was moistened with Ultra-pure water (*Invitrogen*) and gently rubbed over the surface of the eggshell for approximately five minutes (Figure S1a). A sterile scalpel blade was then used to cut the cotton off the swab into a 2.0 mL Safe-lock Lo-Bind *Eppendorf* tube, which was then snap-frozen at -20°C.

Internal sample. 1 mL of Ultra-pure water (*Invitrogen*) was pipetted into the blow-hole of the egg, and rotated for five minutes at room temperature by hand. The liquid was then drained out of the blow-hole into a 1.5 mL Safe-lock Lo-Bind *Eppendorf* tube by inverting the egg. Potentially also eggshell could be rinsed inside with 70% ethanol to ensure any water is removed and the egg is dry for long-term storage. Note that not all eggs had the internal surface sampled because they were too small for the water to drain out of the egg completely. In total 18 eggs had the internal surface sampled.

Eggshell powder. A 0.8 mm drill bit attached to a *Dremel Micro* (via the #483 collet) was moistened with Ultra-pure water (Invitrogen) and used on the lowest power setting to collect eggshell powder by widening the blow-hole, often holding the drill still and gently rotating the egg (Figure S1b). Eggshell powder stuck to the drill bit was rinsed off into 1.4 mL of Ultra-pure water in a 1.5 mL Safe-lock Lo-Bind *Eppendorf* tube. The blow-hole was widened to no more than 2.5X the initial diameter, or to a maximum of 5 mm diameter. The mass of powder collected from each egg was similar (approximately 10-20 mg), but was too small to accurately quantify (Figure S1c).

Internal samples and eggshell powder suspended in water were immediately centrifuged for 10 minutes at maximum speed in a bench-top centrifuge to collect the debris at the bottom of the tube. The

supernatant was then removed and discarded. Samples were then snap-frozen at -20°C. The final diameter of the blow-hole was measured (Table S1, Table S2).

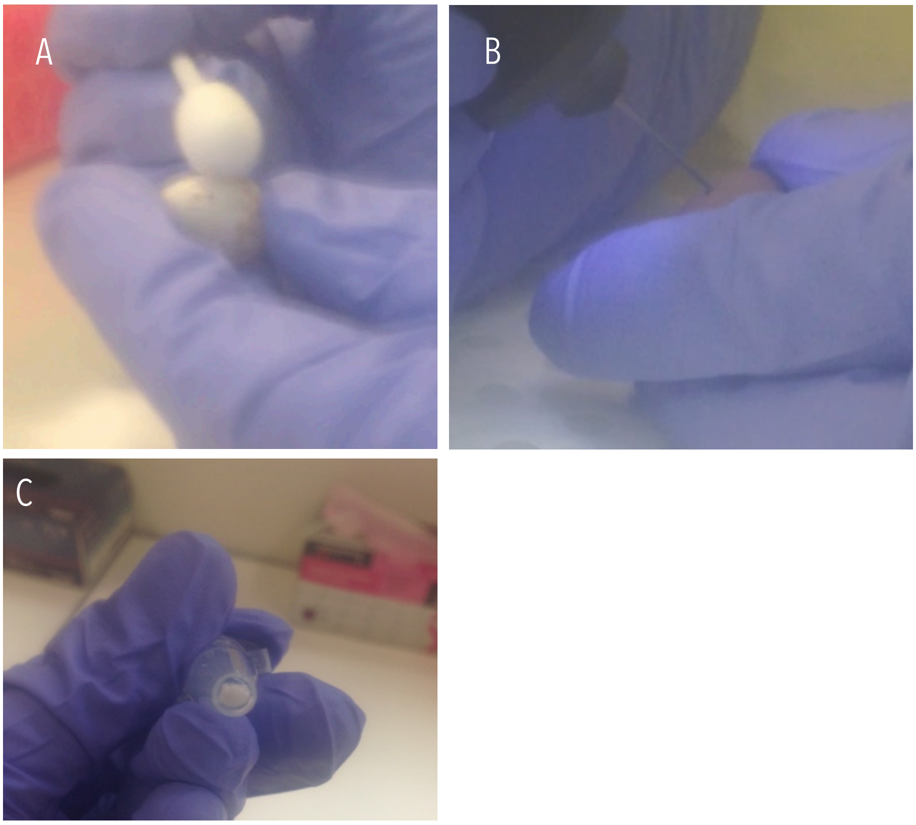

Figure S1 | Photographs of the: **A** swabbing process, **B** drilling process, and **C** approximate amount of powder obtained from drilling (note that in some cases there was perhaps half this amount of powder). Photographs by Alicia Grealy

Other observations. We experimented with several sized and shaped drill bits (Figure S2). Some of these drill bits tended to catch on any loose membrane and tear the eggshell away further; others did not allow the powder to easily be released from the bit into water. A moistened standard 0.8 mm twist drill bit (Figure S2d) proved the gentlest on every egg size while also catching and releasing powder with ease. We also experimented with various-types of self-adhesive templates to reinforce the blow hole prior to drilling and to provide a guide; however, all types of adhesive tended to tear away eggshell in an unpredictable manner, even when the adhesive was low-tack or archival. We therefore do not recommend using any adhesive on the eggshell.

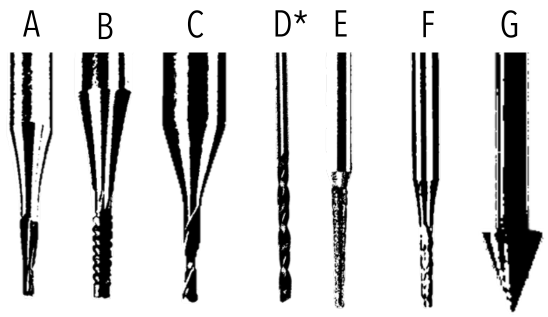

Figure S2 | Various types of drill bits tested to sample eggshell powder: A Tapered, **B** Straight, **C** Carbide down-cut inlay, **D** Plain shank (of varying thickness, but the best was 0.8 mm), **E** Diamond wheel point taper, **F** Carbide grout bit, **G** High speed cutter. The asterisk indicates that this was the best performing drill bit.

SI 1.3 DNA extraction of collection eggshell

All extraction steps were carried out in a designated ultra-clean facility at the ANU (Ecogenomics and Bioinformatics Laboratory) and were extracted in an area separate from the sampling area. 200 ul of a digest buffer containing 2 mg/mL Proteinase K (*Ambion*) in 0.5 M EDTA (*Invitrogen*) was added to eggshell powder and internal samples. 1000 ul of the same digest buffer was added to the swab samples. Samples were incubated with shaking at 1000 rpm overnight at 55°C in a Thermoshaker (*Eppendorf*). Digests were centrifuged at maximum speed for 10 minutes in a bench-top centrifuge to collect cell debris. For the powder and internal samples, the supernatant was removed and placed in a clean 15 mL Falcon tube along with 4 mL of Glocke and Meyer (2017) binding buffer (i.e., 2M Guanidine Hydrochloride, 70% isopropanol, 0.05% Tween-20, and 1:250 v/v *Qiagen* pH indicator in Ultra-pure water). For the swab samples, the supernatant was transferred to a Vivaspin 500 centrifugal concentrated column (MWCO 30 kDa) and concentrated to a volume of 50 ul by centrifuging at 15,000 x *g*, discarding the flow-through. The 50 ul concentrated digest was transferred to a 1.5 mL Safe-lock Lo-Bind Eppendorf tubed and combined with 650 ul of Glock and Meyer (2017) binding buffer. 700 ul of the binding-buffer/digest solution at a time was passed through a MinElute PCR purification silica spin column (*Qiagen*) by centrifugation for 1 minute at 13,000 rpm, discarding the flow-through. 750 ul of PE buffer (*Qiagen*) was passed through the column twice by centrifugation for 1 minute at 13,000 rpm, discarding the flow-through each time. The silica membrane was dried by centrifuging the column for a further 1 minute at 13,000 rpm. The column was placed in a clean 1.5 mL Lo-Bind *Eppendorf* tube with the lid cut off, and allowed to incubate at 37°C for 5 minutes after the addition of 15 ul EB buffer (*Qiagen*) to the silica membrane. DNA was eluted by centrifuging for 1 minute at 10,000 rpm. An additional 15 ul of EB buffer was then passed through the column as above for a total of 30 ul of eluate. Finally, the eluate was passed back through the column as above after a further 5 minutes of incubation at 37°C. The eluate was transferred to a clean 0.5 ml Safe-lock Lo-bind *Eppendorf* tube. 1.5 ul of 1% TE-Tween-20 was added to the extract, which was then stored at -20°C. DNA free extraction controls were included.

SI 1.4 Amplification and sequencing of mini-barcodes

PCR reaction set-up was carried out in a designated ultra-clean facility at the ANU (Ecogenomics and Bioinformatics Laboratory) in designated UV hood inside a physically separate room from the DNA extraction and sampling room. DNA extracts were amplified with two avian-specific mitochondrial 12S rRNA mini-barcodes: 12SAC with a 53 bp insert (Forward 5'-CTGGGATTAGATACCCCACTAT-3', Reverse 5'-GTTTTAAGCGTTTGTGCTCG-3') and 12SAH with a 232 bp insert (Forward 5'-CTGGGATTAGATACCCCACTAT-3', Reverse 5'-CCTTGACCTGTCTTGTTAGC-3') (Cooper 1994), following the methods described by Grealy et al. (2019). The PCR reaction contained reagents in final concentrations of: 1.2 mg/ml BSA, 1X Gold PCR buffer (*Applied Biosystems*), 2.5 mM $MgCl_2$, 0.25 mM dNTPs, 1.25 U Amplitaq Gold DNA polymerase, 0.12X SYBR Green, 0.4 µM of each *IDT* primer, and 2 µl DNA in a final reaction volume of 25 µl. Thermal cycling and all post-PCR procedureds were carried out in another physically separated, post-PCR laboratory. Thermal cycling conditions were: 95°C for 10 min, followed by 50 cycles of 95°C for 30 sec, 54°C (12SAC) or 57°C (12SAH) for 30 sec, 72°C for 45 sec, and a final extension of 72°C for 10 minutes. DNA free PCR negative controls were included, as was a positive control (an extract from fresh tissue of *Chalcites minutillus*; note that the DNA for the positive control was added in a separate facility to eliminate the possibility of cross-contamination from this sample).

The longest amplicon for each sample that was successfully amplified was then amplified in triplicate using fusion primers containing *Illumina* flow-cell binding sites, followed by a custom sequencing adapter and unique multiplexing index upstream of the gene-specific primer. The same PCR reaction conditions were employed as above, but 5 ul of DNA extract was used per reaction, and reactions were performed in duplicate. Duplicate reactions were pooled and purified using SeraPure beads at 1.6X beads ratio, following the manufacturer's instructions, and eluting in 20 ul Ultra-pure water. The DNA

concentration of 1 ul of each amplicon was quantified using the Qubit fluorometer (*Invitrogen*) HiSense kit, following the manufacter's instructions. Amplicons were pooled in approximately equimolar concentrations and purified again as above, eluting in 100 ul EB buffer (*Qiagen*). The molarity of the final library was determined by quantitating 5 ul on with the Qubit HiSense kit (*Invitrogen*), following the manufacturer's instructions, and by running 10 ul on the LabChip GXII fragment analyser using the 5K chip. The library was diluted to 2 nM and sequenced on *Illumina's* MiSeq (single end, Nano 300 cycle v2 kit, no indexing) using a spiked-in custom sequencing primer at the BRF based at ANU.

SI 1.5 Taxonomic identification using mini-barcode sequences

Sequences were de-multiplexed in Geneious Prime 2019.0.4 (Kearse et al. 2012; *Biomatters*) allowing 0 mismatches to the index. Adapters and gene-specific primer sequences were trimmed using the 'Trim Ends' function in Geneious Prime, also allowing 0 mismatches. Sequences were quality filtered, abundance filtered, and chimera filtered using Usearch v.8 and v.9 (Edgar 2010), using the following bash script:

```
#!/bin/bash

#input_folder should already exist and contain all the sequence files in .fastq format.
input_folder="input_seqs"

# output_dir is where you would like the quality filtered data to be written - this folder
should not already exist as it will be created by the script
output_folder="seqs_QF"

maxee="0.5"

echo ""
echo @@@@@@@@@@@@@@@@@@@@@@@@@@@@@@@@@@@@
echo QUALITY CONTROL 1: QUALITY FILTERING
echo @@@@@@@@@@@@@@@@@@@@@@@@@@@@@@@@@@@@
echo ""
mkdir ${output_folder}
echo ""

for filename in ${input_folder}/*.fastq
	do
		echo ""
		echo ~~~~~~~~~~~~~~~~~~~~~~~~~~~~~~~~~~~~~~~~~~~~~~~
		echo input is:
		echo ${filename}
		echo ""
		echo Removing sequences with maxee of:
		echo ${maxee}
		echo""
		usearch8  -fastq_filter  ${filename}  -fastaout  "${output_folder}/$(basename
"$filename" .fastq)_QF.fasta" -fastq_maxee ${maxee}
		echo ""
		echo DONE
		echo ""
		echo ~~~~~~~~~~~~~~~~~~~~~~~~~~~~~~~~~~~~~~~~~~~~~~
	done

echo ""
echo DONE
echo ""
echo XXXXXXXXXXXXXXXXXXXXXXXXXXXXXXXXXXXXXXXXXXXXXXXXXXXXXXX

input_folder2="seqs_QF"
output_folder2="seqs_QF_DEREP"

echo ""
echo @@@@@@@@@@@
echo DEREPLICATE
echo @@@@@@@@@@@
echo ""
mkdir ${output_folder2}
echo ""

for filename in ${input_folder2}/*.fasta
	do
		echo ""
```

```
		echo ~~~~~~~~~~~~~~~~~~~~~~~~~~~~~~~~~~~~~~~~~~~~~~~
		echo FINDING UNIQUE SEQUENCES
		echo input is:
		echo ${filename}
		echo""
		usearch8 -derep_fulllength ${filename} -fastaout "${output_folder2}/$(basename
"$filename" .fasta)_DEREP.fasta" -sizeout
		echo ""
		echo DONE
		echo ""
		echo ~~~~~~~~~~~~~~~~~~~~~~~~~~~~~~~~~~~~~~~~~~~~~~~
	done

echo ""
echo DONE
echo ""
echo XXXXXXXXXXXXXXXXXXXXXXXXXXXXXXXXXXXXXXXXXXXXXXXXXXXXXXX


input_folder3="seqs_QF_DEREP"
output_folder3="seqs_QF_DEREP_CF"

# discard reads with clusters less than this %/100 of the total number of reads e.g. for 0.1%
abundance_cutoff=0.001
# abundance_cutoff="0.01"

echo ""
echo @@@@@@@@@@@@@@@
echo COUNT SEQUENCES
echo @@@@@@@@@@@@@@@
mkdir ${output_folder3}
echo ""

for filename in ${input_folder3}/*.fasta
	do
		echo ""
		echo ~~~~~~~~~~~~~~~~~~~~~~~~~~~~~~~~~~~~~~~~~~~~~~~
		echo COUNTING SEQUENCES
		echo input is:
		echo ${filename}
		echo NUMBER OF SEQUENCES =
	    grep ">" -c ${filename}
		grep ">" -c ${filename} > ${filename}_size.txt
		tail ${filename}_size.txt | awk '{print $1*0.01}' >>
${output_folder3}/$(basename "$filename" .fasta)_minsize.txt
		rm -r ${filename}_size.txt
		echo ""
		echo DONE
		echo ""
		echo ~~~~~~~~~~~~~~~~~~~~~~~~~~~~~~~~~~~~~~~~~~~~~~
	done

echo ""
echo DONE
echo ""
echo XXXXXXXXXXXXXXXXXXXXXXXXXXXXXXXXXXXXXXXXXXXXXXXXXXXXXXX

input_folder3="seqs_QF_DEREP"
output_folder3="seqs_QF_DEREP_CF"

echo ""
echo @@@@@@@@@@@@@@@@@@@@@@@@@@@@@@@@@@@@
echo QUALITY CONTROL 2 – CHIMERAS+DENOISE
echo @@@@@@@@@@@@@@@@@@@@@@@@@@@@@@@@@@@@
echo ""

for filename in ${input_folder3}/*.fasta
	do
		echo ""
		echo ~~~~~~~~~~~~~~~~~~~~~~~~~~~~~~~~~~~~~~~~~~~~~~~
		echo REMOVING CHIMERAS AND DENOISING
		echo input is:
		echo ${filename}
		echo""
		usearch9 -unoise ${filename} -fastaout "${output_folder3}/$(basename "$filename"
.fasta)_CF_DENOISE.fasta" -tabbedout "${output_folder3}/$(basename "$filename"
.fasta)_CF_DENOISE.txt"
		echo ""
		echo DONE
```

```
                echo ""
                echo ~~~~~~~~~~~~~~~~~~~~~~~~~~~~~~~~~~~~~~~~~~~~~~~
        done

echo ""
echo DONE
echo ""
echo XXXXXXXXXXXXXXXXXXXXXXXXXXXXXXXXXXXXXXXXXXXXXXXXXXXXXXX


input_folder4="seqs_QF_DEREP_CF"
output_folder4="seqs_QF_DEREP_CF_AF"

echo ""
echo @@@@@@@@@@@@@@@@@@@@@@@@@@@@@@@@@@@@@@@
echo QUALITY CONTROL 3 – ABUNDANCE FILTERING
echo @@@@@@@@@@@@@@@@@@@@@@@@@@@@@@@@@@@@@@@
echo ""
mkdir ${output_folder4}
echo ""

for filename in ${input_folder4}/*.fasta
        do
                echo ""
                echo ~~~~~~~~~~~~~~~~~~~~~~~~~~~~~~~~~~~~~~~~~~~~~~~
                echo input is:
                echo ${filename}
                echo""
                echo REMOVING UNIQUE READS WITH CLUSTERS CONTAINING LESS THAN:
                cat ${input_folder4}/$(basename "$filename" _CF_DENOISE.fasta)_minsize.txt
                usearch8  -sortbysize  ${filename}  -fastaout  "${output_folder4}/$(basename
"$filename"  .fasta)_AF.fasta"  -minsize  $(cat  "${input_folder4}/$(basename  "$filename"
_CF_DENOISE.fasta)_minsize.txt")
                echo ""
                echo DONE
                rm -r ${input_folder4}/$(basename "$filename" _CF_DENOISE.fasta)_minsize.txt
                echo ""
                echo DONE
                echo ""
                echo ~~~~~~~~~~~~~~~~~~~~~~~~~~~~~~~~~~~~~~~~~~~~~~~~~~~~~~~
        done

echo ""
echo DONE
echo ""
echo XXXXXXXXXXXXXXXXXXXXXXXXXXXXXXXXXXXXXXXXXXXXXXXXXXXXXXX


input5="seqs_QF_DEREP_CF_AF"
output5="seqs_QF_DEREP_CF_AF_TOBLAST"
input6="seqs_QF_DEREP_CF_AF_TOBLAST"

# Removes "=" for the BLAST application

echo ""
echo @@@@@@@@@@@@@@@@@@@@
echo FORMATTING FOR BLAST
echo @@@@@@@@@@@@@@@@@@@@
echo ""
mkdir ${output5}
for filename in ${input5}/*.fasta
        do
                echo Formatting
                echo ${filename}
                cp -v ${filename} ${output5}
        done
for filename in ${input6}/*.fasta
        do
                sed -i '' 's/=/;/g' ${filename}
                echo ""
                echo Complete
                echo ""
        done
echo ~~~~~~~~~~~~~~~~~~~~~~~~~~~~~~~~~~~~~~~~
echo ""
echo ""
```

Filtered, unique sequences were aligned to NCBI's GenBank reference database (Benson et al. 2006) for taxonomic identification in Geneious Prime, using the blastn algorithm (Altschul et al. 1990) with the default parameters and the following options: maximum hits 20, query coverage 100, minimum identity 90, output format 6. BLAST was called as follows:

```
blastn -task blastn -db nt -query input.fasta -out output.txt -perc_identity 90 -qcov_hsp_perc
100 -max_target_seqs 20 -max_hsps 20 -outfmt '6 ssciname qseqid sseqid pident qcovhsp length
evalue bitscore staxid'
```

BLAST searches were performed using the Pearcey Supercomputing Cluster at CSIRO.

For each 12SAC read, taxonomy was initially assigned as follows:

- >99 to 100% sequence similarity to reference across 100% of the query: genus
- >96 to <99% sequence similarity to reference across 100% of the query: family
- 90 to <96% sequence similarity to reference across 100% of the query: order

For each 12SAH read, taxonomy was initially assigned as follows:

- >99 to 100% sequence similarity to reference across 100% of the query: species
- >96 to <99% sequence similarity to reference across 100% of the query: genus
- >95 to <96% sequence similarity to reference across 100% of the query: family
- 90 to <95% sequence similarity to reference across 100% of the query: order

These cut-offs were based on alignments made from every available avian 12SAC and 12SAH sequence on GenBank as of December 2019 for 51 Australian families. Sequences were aligned by family and the intra- and inter-specific pairwise identity was calculated by genus in MEGAX (Kumar et al. 2018; Stecher et al. 2020). The inter-generic pairwise identity was calculated by family in the same way. These identities were averaged to obtain the cut-offs above. Results are summarised in Table S2.

Next, IDs were downgraded to the last common ancestor if:

- There was more than one equally top-scoring hit to different taxa
- The closest Australian relative of the match taxon was **not** also represented in GenBank for that locus
- The second top hit did not share the most recent common ancestor with the top hit compared with lower hits
- More than one plausible read assigned to a different taxon for a sample, a plausible read being one where the appearance of the egg is consistent with the taxon

Finally, IDs were upgraded if:

- Only one species exists within a genus or one genus within a family
- If there is only one Australian taxon within a given taxonomic level
- The egg clearly matches in appearance only one taxon
- The most abundant read is 10X more abundant than any other read in that sample

SI 1.6 Statistical analyses

Amplification, reads pass filter, and plausible ID for each extract was marked as 1 (being present) or 0 (being absent) (see Table S2). For each extract type and amplicon, Spearman's non-parametric correlations were conducted to examine the relationship between each of these binary response variables and the size and thickness of eggshell. In each case, all correlations were statistically insignificant ($p>0.05$). Pairwise non-parametric Mann-Whitney U tests with a Bonferroni correction were conducted to compare whether the plausibility of the ID was related to the extract type and/or amplicon (see Fig. 2 of the main text). Statistical tests were performed in Past3.23 (Hammer et al. 2001).

SI 2.0 Supplementary Results

Table S1 | The pre-existing damage and post-sampling damage of the 51 unregistered eggshell specimens used to hone the sampling techniques.

Table S2 | Molecular identifications of the 45 eggshell specimens sampled for DNA. All hits cover 100% of the query. Only the top BLAST hit for the most abundant sequence variant is shown. For complete BLAST files see the associated DataDryad package.

Note: all BLAST hits, including the most abundant sequence variant, were examined but have not been detailed for brevity (full details can be found in the BLAST files on DataDryad). The number of less-abundant reads is reported. Specimens yielding no ID either returned no BLAST hits above 90% identity across 100% of the query, or returned only one taxon from the swab that the egg morphology did not match. These samples probably had very degraded DNA and only amplified contamination.

Table S3 | Intra- and inter- specific and generic pairwise identities within various avian families for 12SAC and 12SAH mini-barcodes.

Table S4 | The lowest-common ancestor for top blast hits of each sequence variant in samples that had more than one unique filtered read. The percentage of the total filtered reads is provided. For downstream analysis, the most abundant read was considered to be the molecular ID, but as discussed, it can be seen that swabs and internal samples in particular yield many sequences of conflicting identity, therefore supporting the idea that they are not reliable methods for sampling eggshell, even if the most abundant read returns a plausible ID. Complete BLAST files can be found on DataDryad.

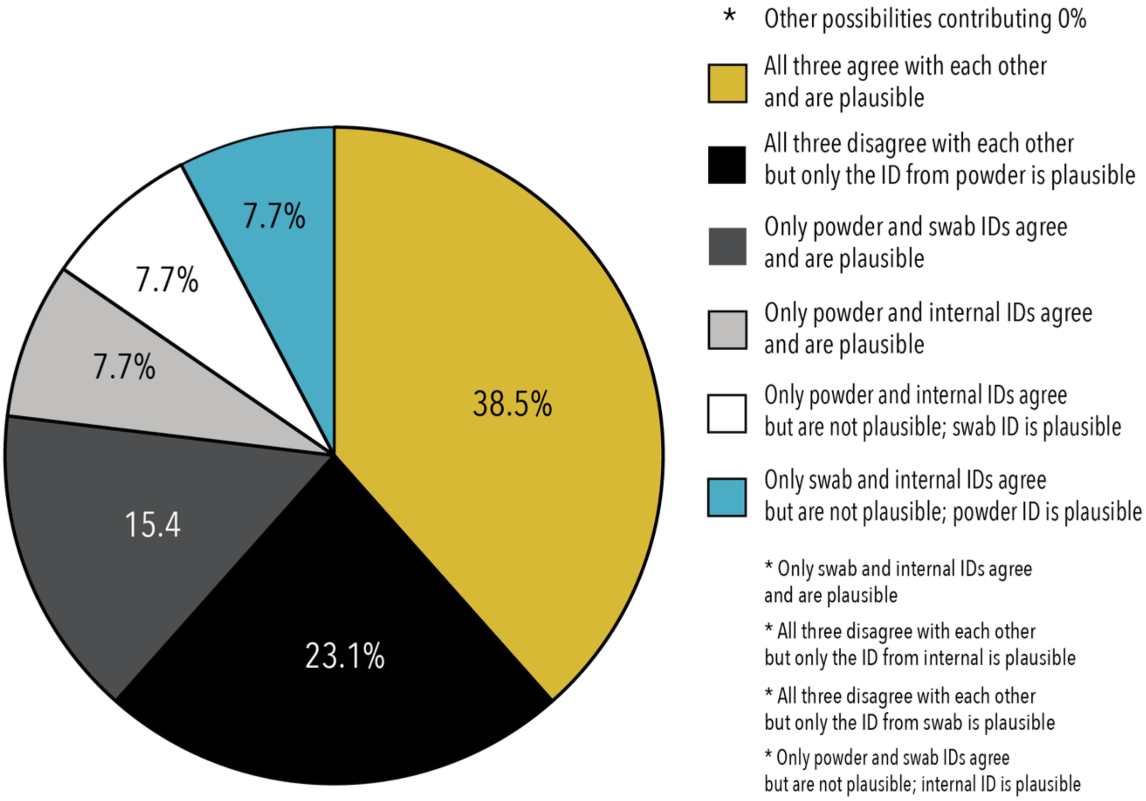

Figure S3 | Agreement among the three extract types in cases where all three produced an identification.

SI 3.0 Supplementary References

Atlas of Living Australia website at http://www.ala.org.au. Accessed March-May 2020.

Altschul, S. F., Gish, W., Miller, W., Myers, E. W., & Lipman, D. J. 1990. Basic local alignment search tool. *Journal of Molecular Biology*, 215, 403–410.

Benson, D. A., Karsch-Mizrachi, I., Lipman, D. J., Ostell, J., & Wheeler, D. L. 2006. GenBank. *Nucleic Acids Research*, *34*, D16–D20.

Cooper, A. 1994. DNA from museum specimens. In: Herrmann, B., Hummel, S., eds. *Ancient DNA*. Springer: New York.

Edgar, R. C. 2010. Search and clustering orders of magnitude faster than BLAST. *Bioinformatics*, 26, 2460–2461.

Stecher, G., Tamura K., and Kumar, S. 2020 Molecular Evolutionary Genetics Analysis (MEGA) for macOS. *Molecular Biology and Evolution,* https://doi.org/10.1093/molbev/msz312

Glocke, I., and Meyer, M. 2017. Extending the spectrum of DNA sequences retrieved from ancient bones and teeth. *Genome Research*, 27, 1230-1237.

Grealy, A., Bunce, M., and Holleley, C. E. 2019. Avian mitochondrial genomes retrieved from museum eggshell. *Molecular Ecology Resources*, 00, 1-11.

Grieve et al. (*manuscript in preparation*). Coevolution with hosts selects for stronger eggshells in brood parasitic cuckoos.

Hammer, Ø., Harper, D. A. T., and Ryan, P. D. 2001. PAST: Paleontological Statistics software package for education and data analysis. *Palaeontologia Electronica*, 4, 9.

Kearse, M., Moir, R., Wilson, A., Stones-Havas, S., Cheung M., Sturrock S., Buxton, S., Cooper, A., Markowitz, S., Duran, C., Thierer, T., Ashton, B., Meintjes, P., Drummond, A. 2012. Geneious Basic: an integrated extendable desktop software platform for the organization and analysis of sequence data. *Bioinformatics*, 28.

Kumar, S., Stecher, G., Li, M., Knyaz, C., and Tamura, K. 2018 MEGA X: Molecular Evolutionary Genetics Analysis across computing platforms. *Molecular Biology and Evolution,* 35, 1547-1549.
